# Intravitreal aflibercept for diabetic macular edema: structural and functional improvements

**DOI:** 10.3389/fmed.2025.1547977

**Published:** 2025-02-19

**Authors:** Chuanhe Zhang, Tianyu Chen, Ru Jia, Di Gong, Zhigao Liu, Changlong Wu, Xiangwen Shu, Fangju Han, Bin Gong

**Affiliations:** ^1^The First Clinical Medical College, Jinan University, Guangzhou, Guangdong, China; ^2^Department of Ophthalmology, Jinan Second People’s Hospital, Jinan, Shandong, China; ^3^Medical Affairs Department, Wuxi Second People’s Hospital, Wuxi, Jiangsu, China; ^4^Shenzhen Eye Hospital, Jinan University, Shenzhen Eye Institute, Shenzhen, Guangdong, China; ^5^Department of Ophthalmology, Jinan Aier Eye Hospital, Jinan, Shandong, China

**Keywords:** diabetic macular edema, aflibercept, central retinal thickness, microperimetry, sensitivity

## Abstract

**Introduction:**

The aim of this study was to evaluate the changes in macular structure and visual function of patients with diabetic macular edema (DME) after intravitreal aflibercept injection.

**Methods:**

Twenty-five patients (43 eyes) diagnosed with DME were included in this study. All patients underwent aflibercept monthly for 3 months. The study’s endpoints included the best corrected visual acuity (BCVA), central retinal thickness (CRT), fovea avascular zone (FAZ) area, vessel density of superficial retinal capillary plexus (SVD), vessel density of deep retinal capillary plexus (DVD), mean light sensitivity (MLS), 2° fixation rate (P1) and 4° fixation rate (P2).

**Results:**

Before treatment and after the third treatment, the LogMAR BCVA was 0.69 ± 0.27 and 0.40 ± 0.18, the CRT was 471.10 ± 159.93 μm and 319.84 ± 113.51 μm, the MLS was 18.14 ± 3.97 dB and 21.68 ± 3.55 dB, P1 was 69 (47, 87)% and 88 (72, 92)%, and P2 was 90 (83, 97)% and 97 (93, 99)%, respectively. After treatment, CRT decreased, BVCA, MLS, and fixation stability improved (all *p* < 0.001). Post-treatment, FAZ area, SVD, and DVD showed no significant changes (all *P* > 0.05). MLS was negatively correlated with LogMAR BCVA and CRT, and positively correlated with P1 and P2.

**Conclusion:**

In short term, aflibercept was effective in reducing CRT and improving BCVA, MLS, and fixation stability in DME patients.

## 1 Introduction

Diabetic retinopathy (DR) is a common and unique microvascular complication of diabetes, while diabetic macular edema (DME) is one of the manifestations of DR, which mainly manifests as the thickening of the retina within the area of two-fold diameters of the optic disk at the central fovea of the macula, and is the major cause of central visual loss in DR patients (1). The pathogenesis of DME involves various factors and is associated with the disruption of the blood-retina barrier, which leads to the leakage of retinal blood vessels and liquid accumulation. In addition, vascular endothelial growth factors (VEGF) also have important roles in the pathogenesis of DME (2). Laser treatment is a conventional treatment for DME, which can effectively reduce edema, but has limited effect on promoting the recovery of visual acuity in patients with vision impairment. Following the continuous advancements of the studies on DME, treatments of DME have also undergone drastic changes. Intravitreal administration of anti-VEGF agents is the preferred initial therapy for DME, with Aflibercept—a fusion protein targeting VEGF-A and Placental Growth Factor—notably enhancing retinal vessel permeability and mitigating macular edema (3–5). Previous studies mainly used optical coherence tomography angiography (OCTA) to examine the changes in macular structures; however, this approach has limited ability to evaluate visual function. Therefore, in the present study, we used microperimetry to evaluate the macular function, which can compensate for the limitations of OCTA examination, and we comprehensively evaluated the treatment effects on DME patients from the aspects of macular structures and function. The clinical significance of the treatment was also investigated in this study.

## 2 Methods

### 2.1 Study design and population

Patients diagnosed with DME in the Jinan Second People’s Hospital between March 2021 and August 2021 were included in this prospective observational study. The inclusion criteria were as follows: (1) adults with Type 2 diabetes, presenting fasting blood glucose levels below 8.0 mmol/L and postprandial levels under 10.0 mmol/L; (2) blood pressure < 150/90 mmHg; (3) those confirmed with macular edema by fundus fluorescein angiography or optical coherence tomography (OCT), and with the central retinal thickness (CRT) of ≥250 μm; and (4) did not undergo ocular relevant treatments before the current treatment or did not receive treatment by intravitreal injection of drugs or pan-retinal photocoagulation within a half year before this treatment.

The exclusion criteria specified: (1) patients with prior eye traumas or other retinal conditions; (2) with severe cataracts that impair fundoscopic assessments; (3) with indications for vitreoretinal surgery, such as vitreous hemorrhage, epiretinal membrane, tractional detachment of retina; (4) subjects previously treated with intravitreal or periocular glucocorticoids; (5) patients with thromboembolic or coagulation disorders, those under anticoagulant therapy (aspirin excluded), or those with significant systemic illnesses; and (6) pregnant or lactating women.

The sample size was calculated using PASS software, with CRT reduction as the primary endpoint. Based on data from a previous study (6), parameters were set at a statistical power of 0.9, *α* = 0.05 (two-tailed test), and a clinically meaningful difference of 73.8 μm. The required sample size was 13 eyes, while this study included 43 eyes, ensuring sufficient power for robust statistical analyses.

This research received approval from the Ethics Committee of Jinan Second People’s Hospital (Approval No.: 20201203), adhering to the Declaration of Helsinki. This research was conducted according to established ethical guidelines.

### 2.2 Procedures

**Figure 1:**
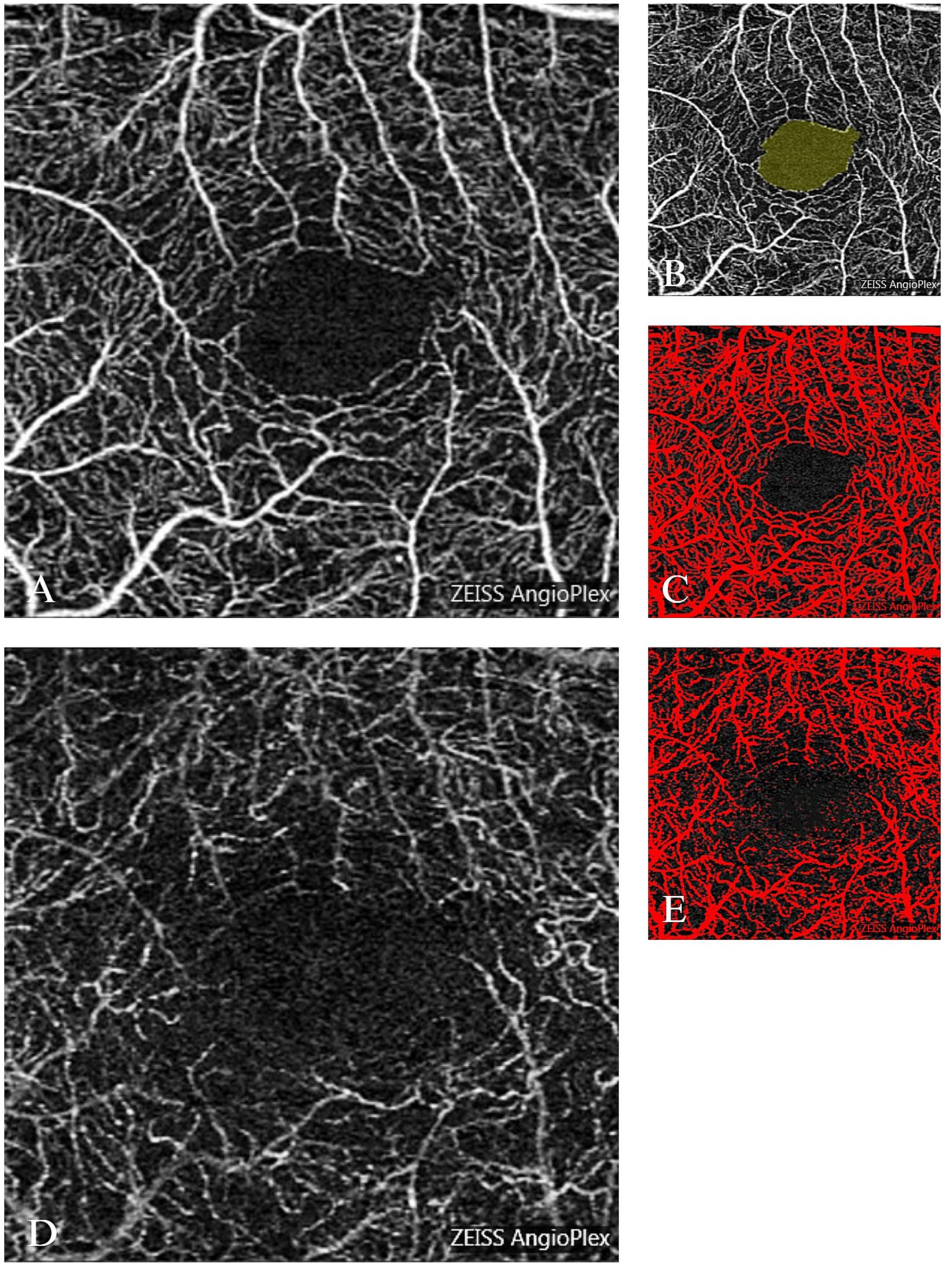
Optical coherence tomography angiography (OCTA) images and measurements of FAZ, SVD, and DVD. **(A)** OCTA image of the SCP. **(B)** FAZ outlined in yellow. **(C)** Superficial capillary outlined in red. **(D)** OCTA image of the DCP. **(E)** Deep capillary outlined in red.

All patients underwent intravitreal injection of aflibercept (IVA) (once per month) continuously for 3 months. The examination was performed with OCT (CIRRUS HD-OCT, Carl Zeiss Meditec Inc., Dublin, CA, United States). Firstly, the OCT system was used for the scanning of the macular area to measure the CRT, after which the AngioPlex mode of OCTA was used for the 3 mm × 3 mm vertical and transverse cross scanning of the macular area, and the analysis program of the system was used for the layer classification of images. This process allowed for the division of the scans into the full-thickness retinal capillary plexus, superficial capillary plexus (SCP), deep capillary plexus (DCP), the foveal avascular zone (FAZ), and the choriocapillaris. To ensure reliability, all OCTA segmented images were carefully reviewed for the accuracy of the automatic segmentation of SCP and DCP. Any errors identified during this review process were manually corrected. The same doctor performed the OCTA examinations of all patients. The Image J software was used to measure the macular FAZ area of SCP, vessel density of superficial retinal capillary plexus (SVD), and vessel density of deep retinal capillary plexus (DVD). Specifically, the FAZ area was manually delineated using the Image J freehand selection tool, while SVD and DVD were quantified using thresholding and binarization techniques to isolate and calculate vessel density within the scanned regions (Figure 1). To ensure the highest level of accuracy, all OCTA images were independently reviewed and analyzed by two experienced ophthalmologists. Any discrepancies were resolved through discussion, and in cases where consensus could not be reached, a senior expert provided the final decision.

The MP-3 microperimetry (NIDEK, Japan) was used for the microperimetry, with the following parameters: the mode of MP1 Macula-10 deg. was selected. The 40 stimulation points were distributed as inner, medium, and outer concentric circles. The diameter of the inner cycle was 2°, and it consisted of 8 points; the diameter of the medium cycle was 6°, and it consisted of 16 points; the diameter of the outer cycle was 10°, and it consisted of 16 points. The background light was white with an illuminance of 31.4 asb. The fixation target utilized was a red cross, approximately 1° in size. The light sensitivity threshold varied between 0 and 34 dB. The mean light sensitivity (MLS) of the retina within the range of 10° (approximately 3 mm), as well as the 2° and 4° fixation rates, were measured. Fixation rates, defined as the proportion of fixation sites within a 2° or 4° diameter circle centered on the fovea to total fixation points (denoted as P1 and P2, respectively), were quantified. The examinations were performed in a dark room, and it was possible to perform a pupil dilation if the pupil was relatively small (<3.3 mm). The doctors helped patients to sit at the microperimetry and asked them to gaze at the fixation target. Next, the patients used split vision to feel the peripheral white stimulation points without tracking the points. Finally, patients were asked to press the response button when spotting any white points. Throughout the procedures, the patients were instructed to maintain fixation; the color photo of the fundus was automatically taken after the processes were completed and overlapped with the photos of microperimetry.

### 2.3 Outcomes

The patients’ indicators, including intraocular pressure, best corrected visual acuity (BCVA), CRT, FAZ area, SVD, DVD, MLS, P1, P2, and other indicators, were collected before and 1 month after each treatment. BCVA was converted into a logarithm of the minimum angle of resolution (LogMAR) for analysis.

### 2.4 Statistical analysis

SPSS 26.0 software (Version 26.0) was used for all the statistical analyses in this study. The Shapiro–Wilk test was employed to assess the normality of the dataset. Normally distributed data were presented as mean ± standard deviation ( $\bar{x}\pm s$ ), while non-normally distributed data were expressed as median and interquartile range. To account for intra-patient correlation between both eyes, generalized estimating equations (GEE) were applied to compare data across different time points before and after treatment, with multiple comparisons corrected using the Bonferroni method. Additionally, GEE was employed to assess correlations among various research indicators before and after each treatment session. GraphPad Prism 9.0 (GraphPad Software Inc.) software was used for figure plotting. Two side *p* < 0.05 was considered statistically significant.

## 3 Results

### 3.1 Baseline characteristics of patients

During the follow-up process, 3 samples had missing values, and the samples containing missing values were directly deleted. A total of 25 patients (43 eyes), including 22 eyes from 12 males and 21 eyes from 13 females, were enrolled in this study. The mean age of patients was 57.33 ± 10.30 years, and the duration of diabetes was 12.35 ± 5.47 years. The baseline characteristics of patients before treatment are shown in Table 1.

**Table 1:** Baseline characteristics of patients.

Baseline characteristics	
Number of patients/eyes	25/43
Age (years) ( $\bar{x}\pm s$ ; range)	57.33 ± 10.30(29–73)
Duration of diabetes (years) ( $\bar{x}\pm s$ ; range)	12.35 ± 5.47(3–25)
Pretreatment LogMAR BCVA ( $\bar{x}\pm s$ ; range)	0.69 ± 0.27(0.22–1.30)
Pretreatment CRT (μm) ( $\bar{x}\pm s$ ; range)	471.10 ± 159.93(250–831)
Pretreatment FAZ area (mm^2^) ( $\bar{x}\pm s$ ; range)	0.37 ± 0.13(0.15–0.69)
Pretreatment SVD (%) ( $\bar{x}\pm s$ ; range)	38.67 ± 4.55(29.09–52.96)
Pretreatment DVD (%) ( $\bar{x}\pm s$ ; range)	40.96 ± 5.72(32.88–57.95)
Pretreatment MLS (dB) ( $\bar{x}\pm s$ ; range)	18.14 ± 3.97(7.20–29.10)
Pretreatment P1 (%) M (P_25_, P_75_)	69(47, 87)
Pretreatment P2 (%) M (P_25_, P_75_)	90(83, 97)

### 3.2 LogMAR BCVA and CRT changes after treatment

**Figure 2:**
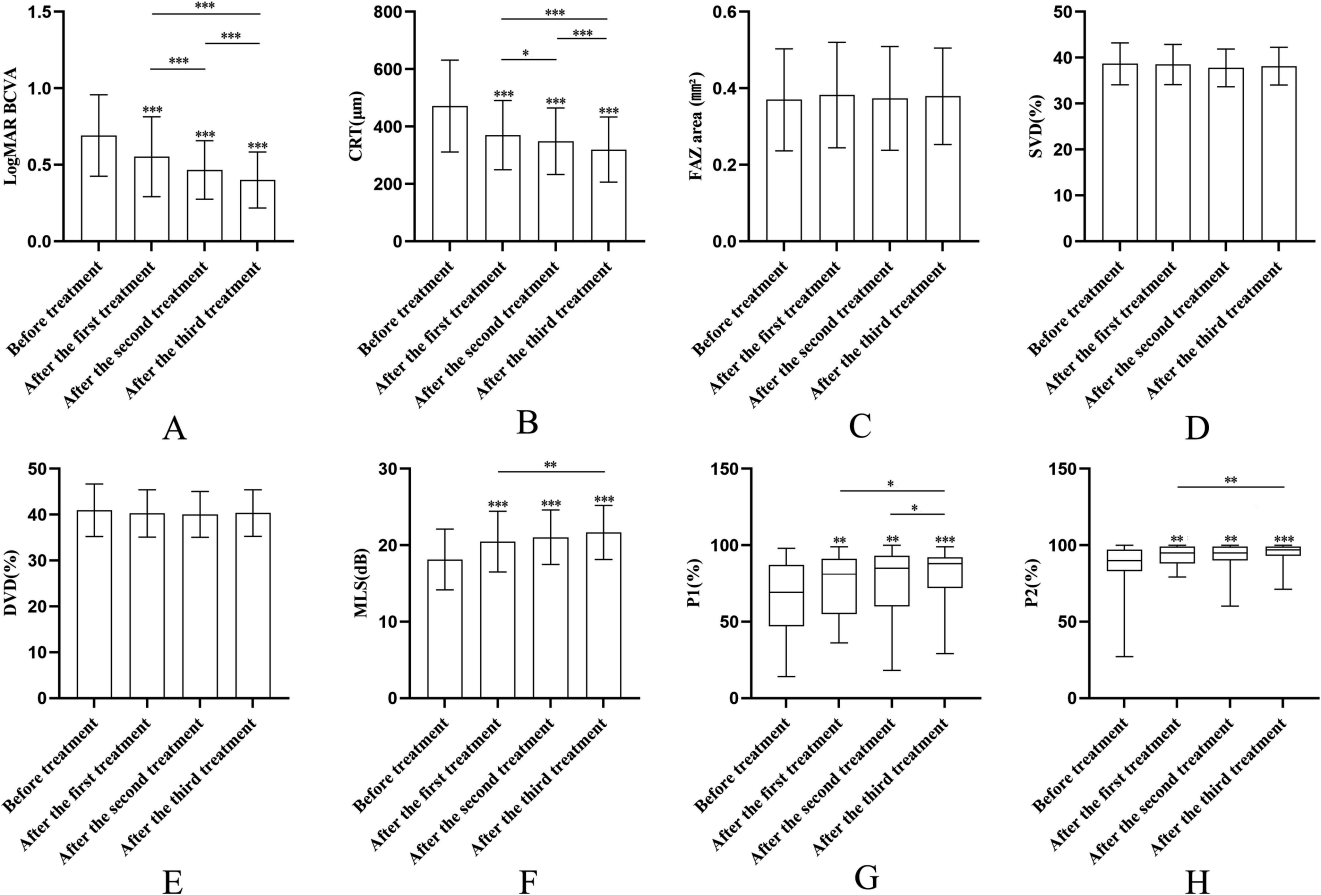
Changes of different indicators in DME patients after anti-VEGF treatment. **(A)** Changes in LogMAR BCVA following treatment. **(B)** Changes in CRT following treatment. **(C)** Changes in FAZ area following treatment. **(D)** Changes in SVD following treatment. **(E)** Changes in DVD following treatment. **(F)** Changes in MLS following treatment. **(G)** Changes in P1 following treatment. **(H)** Changes in P2 following treatment. *p < 0.05, **p < 0.01, ***p < 0.001 (Bonferroni adjustment).

After three anti-VEGF treatments, the respective LogMAR BCVA of patients was 0.55 ± 0.26, 0.47 ± 0.19, and 0.40 ± 0.18, which was significantly lower than before the treatment (all *p* < 0.001), and the respective CRT was 370.30 ± 120.55 μm, 348.93 ± 115.72 μm, and 319.84 ± 113.51 μm, which was also significantly lower than before the treatment (all *p* < 0.001) (Table 2). The LogMAR BCVA and CRT were significantly different among different time points before and after treatment (*all p* < 0.05) (Table 2; Figures 2A,B; Supplementary Table 1).

**Table 2:** Changes in LogMAR BCVA, CRT, FAZ area, SVD, DVD, and MLS in DME patients after anti-VEGF treatment.

Type	LogMAR BCVA	CRT (μm)	FAZ area (mm^2^)	SVD (%)	DVD (%)	MLS (dB)	P1 (%)	P2 (%)
Before treatment	0.69 ± 0.27	471.10 ± 159.93	0.37 ± 0.13	38.67 ± 4.55	40.96 ± 5.72	18.14 ± 3.97	69(47,87)	90(83,97)
After the first treatment	0.55 ± 0.26^***^	370.30 ± 120.55^***^	0.38 ± 0.14	38.53 ± 4.38	40.27 ± 5.18	20.48 ± 3.96^***^	81(55,91)^**^	95(88,99)^**^
After the second treatment	0.47 ± 0.19^***^	348.93 ± 115.72^***^	0.37 ± 0.14	37.78 ± 4.09	40.05 ± 4.99	21.04 ± 3.56^***^	85(60,93)^**^	95(90,99)^**^
After the third treatment	0.40 ± 0.18^***^	319.84 ± 113.51^***^	0.38 ± 0.13	38.29 ± 3.99	40.34 ± 5.08	21.68 ± 3.55^***^	88(72,92)^***^	97(93,99)^***^
Wald *χ*^2^	112.616	68.407	3.849	5.332	4.823	75.079	34.227	22.008
*p*-value	<0.001	<0.001	0.278	0.149	0.185	<0.001	<0.001	<0.001

*p*-values for the overall comparison of indicators among different time points before and after treatment; ^**^P < 0.01, ^***^P<0.001 (Bonferroni adjustment), comparison with the baseline level.

### 3.3 FAZ area, SVD, and DVD changes after treatment

After three anti-VEGF treatments, the respective FAZ area was 0.38 ± 0.14 mm^2^, 0.37 ± 0.14 mm^2^, and 0.38 ± 0.13 mm^2^, the respective SVD was (38.53 ± 4.38)%, (37.78 ± 4.09)%, and (38.29 ± 3.99)% and the respective DVD was (40.27 ± 5.18)%, (40.05 ± 4.99)%, and (40.34 ± 5.08)%. However, there was no statistically significant difference in FAZ area, SVD, and DVD at different time points before and after treatment (*p* = 0.278, *p* = 0.149, *p* = 0.185) (Table 2; Figures 2C–E; Supplementary Table 1).

### 3.4 MLS and P1/P2 changes after treatment

**Figure 3:**
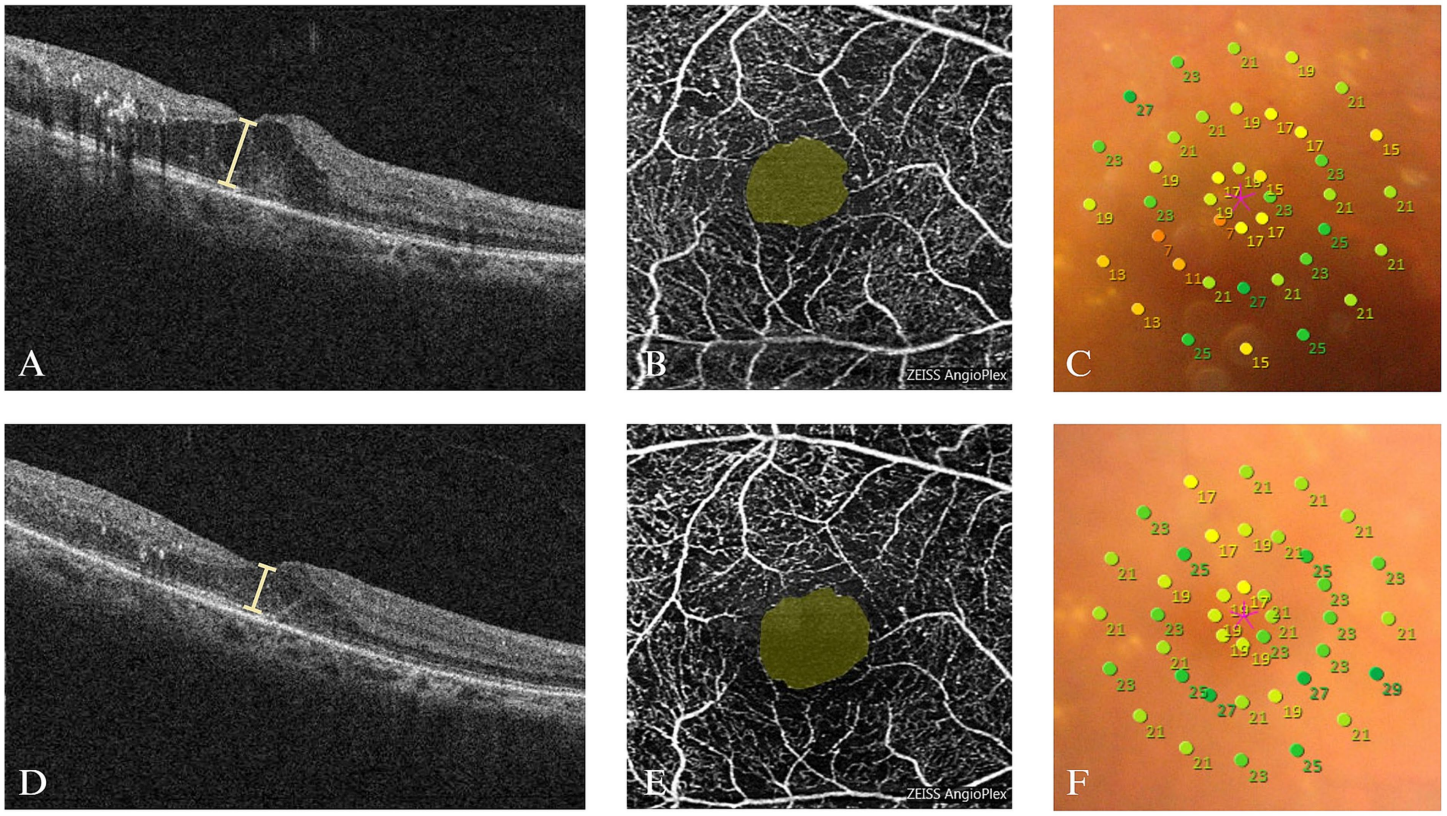
Examination findings of the right eye of a DME patient (female, 58 years old) before and after the third treatment. **(A)** The OCT image of the macula before treatment showing the cystoid macular edema; the CRT was 365 μm. **(B)** OCTA image before treatment showing the destruction of the arch ring and non-perfusion areas in the superficial capillary plexus. **(C)** A microperimetry image of the macular area in diameter of 10° before treatment showing the reduction of local retinal light sensitivity; the MLS was 19.3 dB. **(D)** Macular OCT image after treatment showing that macular edema was reduced compared to before; the CRT was 268 μm. **(E)** OCTA image after treatment showing that the superficial capillary plexus did not significantly change compared to before, and signal masking was found in some regions. **(F)** A microperimetry image of the macular area in diameter of 10° after treatment showing that the local retinal light sensitivity increased compared to before; the MLS was 21.7 dB.

The respective MLS was 20.48 ± 3.96 dB, 21.04 ± 3.56 dB, and 21.68 ± 3.55 dB after three anti-VEGF treatments, which were all significantly increased compared with before the treatment (all *p* < 0.001). No significant change was observed between the first and second treatments (*p* = 1.000) or between the second and third treatments (*p* = 0.081), but a significant increase occurred after the third treatment compared to the first (*p* = 0.007) (Table 2; Figure 2F; Supplementary Table 1). Both P1 and P2 increased significantly after the treatment compared with before the treatment (*all p* < 0.01) (Table 2; Figures 2G,H; Supplementary Table 1). Pre- and post-treatment evaluations of DME patients utilized OCT, OCTA, and microperimetry, as depicted in Figure 3.

### 3.5 Correlation analysis of factors

GEE analysis revealed that, before treatment, LogMAR BCVA showed a positive correlated with CRT (*β* = 241.173, *p* = 0.004), and negative correlations with MLS (*β* = −10.979, *p* < 0.001), P1 (*β* = −37.277, *p* < 0.001), and P2 (*β* = −27.196, *p* = 0.005). It was insignificantly correlated with FAZ area, SVD, and DVD (all *P* > 0.05). CRT was negatively correlated with MLS (*β* = −0.011, *p* < 0.001) and P1 (*β* = −0.070, *p* < 0.001), and insignificantly correlated with FAZ area, SVD, DVD, and P2 (all *P* > 0.05). MLS was positively correlated with P1 (*β* = 2.910, *p* < 0.001) and P2 (*β* = 1.711, *p* < 0.001), negatively correlated with FAZ area (*β* = −8.354, *p* = 0.009), and insignificantly correlated with SVD and DVD (all *P* > 0.05) (Table 3).

**Table 3:** Correlation analysis of research indicators before and after each treatment using generalized estimating equations (GEE).

Research indicators		Before treatment		After the first treatment		After the second treatment		After the third treatment	
		*β*	*p*-value	*β*	*P*-value	*β*	*P*-value	*β*	*P*-value
LogMAR BCVA	CRT	241.173	0.004	153.950	0.093	94.363	0.184	246.882	0.001
	FAZ area	0.010	0.849	0.010	0.812	0.039	0.535	0.012	0.857
	SVD	−1.441	0.679	−3.035	0.156	−1.140	0.696	−3.926	0.124
	DVD	0.938	0.722	−0.587	0.790	−4.644	0.123	−2.112	0.511
	MLS	−10.979	<0.001	−10.364	<0.001	−10.953	<0.001	−14.355	<0.001
	P1	−37.277	<0.001	−10.290	0.356	−26.446	0.007	−33.419	<0.001
	P2	−27.196	0.005	−5.742	0.035	−14.422	0.005	−11.268	0.020
CRT	FAZ area	0.000	0.682	0.000	0.898	0.000	0.228	0.000	0.054
	SVD	−0.009	0.090	−0.003	0.427	−0.002	0.537	0.000	0.946
	DVD	−0.005	0.502	0.002	0.815	−0.002	0.723	−0.002	0.807
	MLS	−0.011	<0.001	−0.010	0.032	−0.009	0.162	−0.012	0.026
	P1	−0.070	<0.001	0.019	0.333	−0.024	0.266	−0.062	0.058
	P2	−0.043	0.065	−0.008	0.386	−0.026	0.163	−0.020	0.218
FAZ area	SVD	−0.439	0.919	−9.051	0.034	−0.435	0.925	1.436	0.805
	DVD	−8.759	0.099	−2.351	0.685	−0.247	0.961	2.764	0.624
	MLS	−8.354	0.009	−9.303	0.039	−2.817	0.469	−3.741	0.304
	P1	−49.465	0.057	−93.111	<0.001	1.999	0.966	−49.868	<0.001
	P2	−16.560	0.138	−27.199	<0.001	−9.622	0.320	−16.937	<0.001
SVD	DVD	0.750	<0.001	0.539	<0.001	0.451	0.006	0.453	0.019
	MLS	0.196	0.056	0.437	<0.001	0.305	0.010	0.338	<0.001
	P1	1.815	0.039	1.057	0.068	0.088	0.896	0.366	0.377
	P2	0.825	0.149	0.314	0.098	0.254	0.279	0.018	0.905
DVD	MLS	0.053	0.539	0.247	0.034	0.165	0.158	0.211	0.074
	P1	0.385	0.598	0.293	0.522	0.333	0.422	−0.080	0.868
	P2	0.216	0.641	0.038	0.849	0.172	0.432	−0.111	0.617
MLS	P1	2.910	<0.001	0.861	0.131	1.591	0.004	2.252	0.002
	P2	1.711	0.016	0.239	0.242	1.128	0.007	0.858	0.013
P1	P2	0.458	<0.001	0.247	<0.001	0.343	<0.001	0.341	<0.001

After the third treatment, LogMAR BCVA was still positively correlated with CRT (*β* = 246.882, *p* = 0.001), and negatively correlated with MLS (*β* = −14.355, *p* < 0.001), P1 (*β* = −33.419, *p* < 0.001), and P2 (*β* = −11.268, *p* = 0.020), but had no significant correlation with FAZ area, SVD, and DVD (all *P* > 0.05). CRT was negatively correlated with MLS (*β* = −0.012, *p* = 0.026), and insignificantly correlated with FAZ, SVD, DVD, P1, and P2 (all *P* > 0.05). MLS was positively correlated with SVD (*β* = 0.338, *p* < 0.001), P1 (*β* = 2.252, *p* = 0.002), and P2 (*β* = 0.858, *p* = 0.013), and insignificantly correlated with FAZ area and DVD (all *P* > 0.05) (Table 3).

## 4 Discussion

The present study included DME patients who underwent treatment with IVA, finding that anti-VEGF treatment could effectively reduce the CRT, increase the retina’s light sensitivity, and improve visual acuity. The findings on macular structures and function provided more valuable evidence for the safety and effectiveness of aflibercept for the treatment of DME.

The study demonstrated significant improvements in visual acuity and reductions in CRT post-treatment, aligning with findings by Qin et al. (7). The VISTA/VIVID-DME study showed that treating DME with anti-VEGF could significantly benefit BCVA and reduce CRT (8), which is in line with our results. These findings demonstrated that anti-VEGF treatment could significantly reduce the permeability of retinal vessels, reduce the exudation of liquids in blood vessels, improve the blood-retina barrier, and alleviate macular edema. In addition, the CRT of very few patients in this study was significantly reduced; however, the visual acuity did not significantly improve in this study after anti-VEGF treatment, which could be associated with the injuries of the retinal photoreceptor cell.

The FAZ is surrounded by the continuous retinal capillary plexus with no capillary structures. It is also a very important area for forming fine visual function, where the changes in morphology and density of surrounding capillaries could reflect the degree of macular ischemia, being closely associated with various retinal diseases, especially retinal vascular diseases (9). Occlusion of capillaries surrounding the macula could induce FAZ disruption and increase the area. Thus, the FAZ area in DR patients is larger than in healthy people, while the SVD and DVD are both reduced (10, 11). Indicators such as FAZ area, SVD, and DVD could visually reflect the retinal microcirculation and be used to further predict the progression of visual acuity and monitor the treatment responses (12). In this study, the FAZ area, SVD, and DVD in patients who underwent IVA treatment were not significantly different at different time points compared with before treatment. Similarly, Mirshahi et al. (13) found no substantial changes in FAZ areas or vascular density post-bevacizumab injections. Busch et al. (14) reported that after IVA for the treatment of DME (time points for observation were 3–25 months), the retinal microvascular density also did not change, which is consistent with our findings. We assumed that the blood glucose level in diabetes patients remains elevated, whose effects, along with the oppression from edema, could lead to irreversible injuries to the retinal vascular structures and could induce vascular obstruction, arch ring destruction, FAZ enlargement, and vascular density reduction. It was hypothesized that while anti-VEGF treatment may reduce fluid exudation, it does not effectively clear blocked blood vessels nor enhance macular blood perfusion. Nonetheless, this study documented enhanced regional blood perfusion in certain patients following anti-VEGF therapy. This improvement is likely linked to the rapid resolution of edema. When the macular edema was very severe, the exuded liquids could press the retinal blood vessels, thus masking the part of blood flow signals on OCTA; while after edema they disappeared, the masking effect reduced, and the signals reappeared again; however, the states of blood perfusion did not actually change. Under such circumstances, even the macular edema disappeared, and the visual benefits were generally limited. These findings suggest that using OCTA to examine the FAZ region and vascular density has important significance for early observation and early treatments of patients with DME.

There are still debates on the influence of anti-VEGF treatment on macular microcirculation in patients with diabetic macular edema. Mastropasqua et al. (15) found that within the 5 months’ of follow-up of DME patients who underwent IVA, SVD and DVD were significantly increased. Hsieh et al. (16) used OCTA to examine the changes of biomarkers in DME patients treated with ranibizumab, finding that the vascular density close to the central fovea was increased compared to before the realizations of 3 treatments. Still, some studies suggested that anti-VEGF treatment could worsen retinal ischemia. For instance, in their retrospective study, Feucht et al. (17) treated patients with macular edema-induced non-proliferative diabetic retinopathy or branch retinal vein occlusion by intravitreal injection of bevacizumab, finding that the FAZ area increased after 6–8 weeks, which was indicative of the worsened retinal ischemia. Yet, the investigators speculated that such changes could be transient. As we could not evaluate the potential changes in retinal vascular density and FAZ in untreated eyes, it remains unclear whether IVA could prevent the progression of macular ischemia. Nonetheless, previous studies have demonstrated that IVA would at least not worsen the blood flow of the retina in most patients with DME. These findings suggested that the major cause of macular edema alleviation in the short-term treatment of DME by anti-VEGF could be the alleviation of retinal vascular exudation, while the retinal vascular occlusion did not significantly improve; nevertheless, the perfusion defect in macular vessels also did not worsen.

Currently, the visual functions of DME patients are generally evaluated by central visual acuity. However, the central visual acuity acquired by the visual acuity chart is not suitable for sophisticated quantitative evaluation of visual function (18–20). While morphological changes are generally used as indicators for treatments of DME, the change in visual function is the major concern of patients. MP-3 microperimetry could be used to automatically evaluate the retina’s sensitivity in the macular area and quantify the visual function of the central fovea of the macula. Retinal sensitivity, as compared to central visual acuity, offers a more precise assessment of the nuances in macular visual function alterations. In addition, P1 and P2 could well reflect the stability of fixation of patients and reflect the quality of vision (21–23). Our results revealed that MLS, P1, and P2 increased significantly after treatment. In their study, Xu et al. (24) evaluated the retinal structures and visual function changes in DME patients 1 year after IVA, finding that the MLS at the central fovea was significantly improved, which was closely associated with the beneficial effects on BCVA. Ichio et al. (25) and Malagola et al. (26) treated DME patients with anti-VEGF (once per month) for 3 months and found that the MLS of the retina significantly increased compared with the baseline level. In addition, the thickness of the macular central fovea was significantly correlated with the MLS of the retina, which is in agreement with the findings of this study, demonstrating that intravitreal injection of anti-VEGF agents could significantly improve retinal sensitivity. However, several studies have demonstrated that the retinal sensitivity after anti-VEGF treatment showed no statistically significant change, which could be associated with the relatively high standard deviations and a limited number of eyes evaluated in the corresponding studies (27). Seidensticker et al. (28) found that the fixation stability of DME patients after intravitreal injection of ranibizumab significantly improved compared to before treatment, but the retinal sensitivity did not significantly change. The investigators speculated that the fixation stability could be used as an early indicator for evaluating the changes in visual function. These findings were not in agreement with the results of this study, which might be due to the difference in the length of disease duration of DME in the patients. Specifically, patients in Seidensticker’s study mainly had long disease duration, and their anatomical structures of the retina were already significantly changed. Although CRT reduced and central visual acuity improved, the retina surrounding the central fovea showed no significant functional improvement. An interesting observation in our study was that the changes in MLS between the first and second treatments, as well as between the second and third treatments, were not statistically significant. We believe this may be attributed to several factors. First, early structural recovery often precedes functional improvement. Functional recovery, such as changes in MLS, may take longer to manifest as it depends on the restoration of photoreceptor function, which may not progress linearly with structural changes. Second, subsequent treatments may require more time or cumulative effects to achieve further enhancements in retinal sensitivity. Additionally, inter-individual variability in baseline retinal damage, such as differences in photoreceptor integrity, may lead to varying responses among patients. Lastly, the observed changes during this interval might have been influenced by the sample size and variability, which could limit the statistical power to detect smaller but potentially meaningful improvements.

The findings of the present study demonstrated that MLS before and after treatment was associated with visual acuity but also with CRT, which was in agreement with the results of a study by Vujosevic et al. (29). Previous studies have demonstrated that the macular central fovea thickness measured by OCT is significantly associated with BCVA (30, 31), which is consistent with our results. Several researchers divided the macular area into 9 regions, after which they explored the association between microperimetry and OCTA findings. Their findings showed that the retinal sensitivity of central fovea on the temporal side was moderately associated with vascular density, while the associations in the other 8 regions were not statistically significant (32). In this study, MLS of the retina in the 10° was measured, and the findings showed that MLS correlated with SVD and DVD only after treatment. In contrast, before treatment, LogMAR BCVA showed a negative correlation with both P1 and P2, whereas MLS exhibited a positive correlation with these parameters. However, these correlations changed after treatment, which we believe may be attributed to several factors. One possible explanation is that anti-VEGF therapy, such as aflibercept, primarily reduces macular edema and improves CRT in the short term, resulting in rapid improvements in BCVA and MLS. In contrast, fixation stability (P1 and P2) may recover more gradually, as it relies on more complex interactions between retinal structure and function. Another contributing factor could be individual differences, such as the severity of macular edema or the extent of retinal damage, which might influence how patients respond to treatment. These variations could have contributed to the observed changes in correlation patterns. While extending the follow-up period would undoubtedly provide deeper insights, our current findings emphasize the short-term effectiveness of aflibercept in improving retinal structure and function. We will aim to incorporate longer follow-up durations in future studies to further clarify these changes in retinal structure and function. Moreover, we propose retinal sensitivity and fixation stability as valuable metrics for assessing DME treatment efficacy.

The present study has some limitations. The study’s sample size was relatively small, so future studies with larger sample sizes are needed to further verify and confirm the reported findings. The follow-up time of this study was relatively short. The retinal sensitivity and vascular density in this study were calculated as the mean values of the measured macular regions, while the visual function and blood flow changes in some small regions might be neglected. In the current study, we used the latest generation of MP-3 microperimetry. The differences in parameters and internal programs of different devices could also influence the findings.

## 5 Conclusion

In conclusion, treating DME patients with aflibercept within a short time led to reduced retinal thickness, increased retinal sensitivity, and improved visual acuity. Furthermore, improvements in visual function are associated with changes in retinal structure, and retinal sensitivity and fixation stability could be used as reliable supplementary functional parameters for evaluating the processes of DME treatment. Yet, more studies are needed to further investigate the influence of anti-VEGF treatment on retinal microcirculation and the correlation between macular structures and visual function.

## Data Availability

The original contributions presented in the study are included in the article/Supplementary material, further inquiries can be directed to the corresponding author.
