# Supplementary material for: Intravitreal aflibercept for diabetic macular edema: structural and functional improvements
Source: Front Med (Lausanne). 2025 Feb 19;12:1547977. doi: 10.3389/fmed.2025.1547977 (PMC11880015; doi:10.3389/fmed.2025.1547977)
Supplement: Supplementary file 1 [file Table_1.docx]

**Supplementary Table 1.** Significance levels of study parameters at different treatment time points (after Bonferroni adjustment)

| Comparison at different treatment time points | LogMAR BCVA | CRT | FAZ area | SVD | DVD | MLS | P1 | P2 |
| --- | --- | --- | --- | --- | --- | --- | --- | --- |
| Before treatment vs. After the first treatment | ＜0.001 | ＜0.001 | 0.491 | 1.000 | 1.000 | ＜0.001 | 0.006 | 0.002 |
| Before treatment vs. After the second treatment | ＜0.001 | ＜0.001 | 1.000 | 0.617 | 0.821 | ＜0.001 | 0.005 | 0.002 |
| Before treatment vs. After the third treatment | ＜0.001 | ＜0.001 | 1.000 | 1.000 | 1.000 | ＜0.001 | ＜0.001 | ＜0.001 |
| After the first treatment vs. After the second treatment | ＜0.001 | 0.043 | 1.000 | 0.833 | 1.000 | 1.000 | 1.000 | 1.000 |
| After the first treatment vs. After the third treatment | ＜0.001 | ＜0.001 | 1.000 | 1.000 | 1.000 | 0.007 | 0.015 | 0.007 |
| After the second treatment vs. After the third treatment | ＜0.001 | ＜0.001 | 1.000 | 0.137 | 1.000 | 0.081 | 0.013 | 0.132 |
